# Materials for Production of High and Ultra-High Performance Concrete: Review and Perspective of Possible Novel Materials

**DOI:** 10.3390/ma14154304

**Published:** 2021-07-31

**Authors:** Markssuel Teixeira Marvila, Afonso Rangel Garcez de Azevedo, Paulo R. de Matos, Sergio Neves Monteiro, Carlos Maurício Fontes Vieira

**Affiliations:** 1 LAMAV—Advanced Materials Laboratory, UENF—State University of the Northern Rio de Janeiro, Av. Alberto Lamego, 2000, Campos dos Goytacazes 28013-602, Brazil; markssuel@hotmail.com (M.T.M.); veira@uenf.br (C.M.F.V.); 2 LECIV—Civil Engineering Laboratory, UENF—State University of the Northern Rio de Janeiro, Av. Alberto Lamego, 2000, Campos dos Goytacazes 28013-602, Brazil; 3 Department of Civil Engineering, UFSC—Federal University of Santa Catarina, Rua João Pio Duarte Silva, 205, Florianópolis 88040-900, Brazil; paulorm.matos@gmail.com; 4 Coordenadoria Acadêmica, UFSM—Federal University of Santa Maria, Rodovia Taufik Germano, 3013, Cachoeira do Sul 96503-205, Brazil; 5 Department of Materials Science, IME—Military Institute of Engineering, Square General Tibúrcio, 80, Rio de Janeiro 22290-270, Brazil; snevesmonteiro@gmail.com

**Keywords:** high-performance concrete, ultra-high-performance concrete, fibers, mechanical strength, alkali-activated cement, geopolymers

## Abstract

This review article proposes the identification and basic concepts of materials that might be used for the production of high-performance concrete (HPC) and ultra-high-performance concrete (UHPC). Although other reviews have addressed this topic, the present work differs by presenting relevant aspects on possible materials applied in the production of HPC and UHPC. The main innovation of this review article is to identify the perspectives for new materials that can be considered in the production of novel special concretes. After consulting different bibliographic databases, some information related to ordinary Portland cement (OPC), mineral additions, aggregates, and chemical additives used for the production of HPC and UHPC were highlighted. Relevant information on the application of synthetic and natural fibers is also highlighted in association with a cement matrix of HPC and UHPC, forming composites with properties superior to conventional concrete used in civil construction. The article also presents some relevant characteristics for the application of HPC and UHPC produced with alkali-activated cement, an alternative binder to OPC produced through the reaction between two essential components: precursors and activators. Some information about the main types of precursors, subdivided into materials rich in aluminosilicates and rich in calcium, were also highlighted. Finally, suggestions for future work related to the application of HPC and UHPC are highlighted, guiding future research on this topic.

## 1. Introduction

With the advancement of cement science and technology and due to the necessity for slender and bolder structures, conventional concrete (CC), also named as normal strength concrete [1], no longer meets the requirements for the execution of these works. In this context, other concretes and other cementitious mixtures, with properties superior to CC, emerged to meet this need. This is the case for high-strength concrete (HSC) [2], high-performance concrete (HPC) [1,3] and, more recently, ultra-high performance concrete (UHPC) [3,4].

Another important aspect of using HPC and UHPC is the environmental issue, as these concretes provide lower values of binder intensity (*bi*). This index measures the total amount of binder needed to provide one unit of a given performance indicator, for example, compressive strength in MPa, as per Equation (1). In this sense, the application of these types of concrete provides an ecological gain, due to the reduction in cement consumption to reach the same level of compressive strength [5,6].

(1) $$bi=\frac{b}{\sigma }$$

where *b* is the total consumption of binder materials (kg·m^−3^) and *σ* is the compressive strength (MPa) at 28 days.

It is very common to confuse these terms, due to the absence of technical standards that provide satisfactory definitions for these materials. Some standards for concrete structures, such as NBR 8953 [7], NBR 6118 [8], ACI 363 [9], ACI 318 [10], and BS EN 1992 [11], differentiate two classes of concrete, as a function of characteristic compressive strength (f_ck_). For instance, class I concretes have a compressive strength between 20 and 50 MPa, while class II concretes have a strength between 55 and 100 MPa. It can be considered that class I concretes be associated with the CC while class II concretes are related to HSC.

While the definition of HSC is based only on compressive strength, the definition of HPC and UHPC is related to performance, which includes not only mechanical strength but also workability, aesthetics, finish, integrity, and durability [12,13]. Despite not being unanimous, a satisfactory definition for the HPC is that this concrete presents a compressive strength equivalent to the HSC, that is, f_ck_ above 50 or 55 MPa [2,14], but presents a workability equivalent to self-compacting concrete (SCC), that is, a spread between 455 to 810 mm by the slump-flow test [1,15,16]. Regarding the water/binder factor (w/b), some authors suggest that this factor should be less than 0.40 [17,18,19], contrary to what is observed in conventional concretes, where the w/b factor ranges from 0.45 to 0.65 [20,21,22]. Regarding cement consumption, it is usual for it to be between 400 to 700 kg/m^3^ [23,24,25,26], while for CC, the cement consumption is usually between 260 to 380 kg/m^3^ [25,27,28].

The UHPC, on the other hand, presents even greater requirements. Some authors suggest a minimum strength of 120 MPa [13,29], while others stipulate a minimum of 150 MPa [30,31], with a fluidity equivalent or greater than HPC, in addition to low porosity. In theory, a concrete that presents a strength above class II could be considered a concrete with a mechanical performance superior to HSC or HPC. In other words, these materials would compose a kind of strength class III, where the UHPC fits. These properties are achieved using a w/c ratio between 0.2 to 0.3 [32,33] associated with a very high cement consumption, around 800 to 1000 kg/m^3^ [34,35]. In addition, UHPC is usually produced without the use of coarse aggregates, which theoretically turns it into a mortar, allowing the evaluation of workability to be carried out by the flow table test. Some authors recommend that flow table measurements be greater than 260 mm, without the application of blows to the material [36]. All this information is summarized in Table 1, which presents the main characteristics of the concretes discussed in this literature review.

Another way to understand the difference between the concretes presented in Table 1, from the point of view of compressive strength. This is one of the reasons for the need to apply fibers in UHPC, which will be further discussed in this paper. In addition to conventional (i.e., steel and polymeric) fibers, the search for alternative natural fibers, such as basalt, sisal, and banana fibers has grown in recent years (detailed in Section 3.3) mainly due to their low cost and good fiber-matrix bond. Finally, despite the great interest in the application of fibers in HPC and UHPC, few standards are available to specify it [37].

The main applications of HPC and UHPC initially were in the construction of high-rise residential and/or commercial buildings, mainly between the 60 and 80 s. Examples are the Lake Point Tower and Water Tower Place buildings, in Chicago—the USA, built, respectively, in 1965 and 1970 [23,38]. Later, these classes of concrete started to be applied more frequently in infrastructure works, through the construction of bridges, viaducts, and other special buildings of art [39,40,41]. Since then, HPC and UHPC have been also applied to road pavements, industrial floors, and underground buildings [42,43]. More broadly, these types of concretes can be applied in any works requiring high compression loads as well as structures subjected to aggressive environments that need high durability and in cases of emergency or recovery works [12,13,44]. However, the cost must be taken into account when analyzing the feasibility of the building construction, since in general high-performance concrete is more expensive than conventional concrete [45,46].

In this context, the objective of this literature review is to present some concepts related to the main materials used for the production of HPC and UHPC. The materials named in this research as “classics” will be highlighted, as they are the same ones traditionally used to elaborate the CC. In addition, it is also an objective of this review to identify new materials used to obtain (ultra) high-performance concretes; in particular through the inclusion of fibers and the use of alkali-activated cement.

## 2. Classic Components Used for HPC and UHPC Production

In this section, the main materials that make up the structure of HPC and UHPC will be described. These materials are named classics as they are also used for CC production. These are ordinary Portland cement (OPC), mineral additions, aggregates, and chemical additives.

### 2.1. Ordinary Portland Cement (OPC)

The OPC used for the production of HPC and UHPC can be the same for the production of CC. However, several authors recommend the use of OPC with higher clinker contents and lower amounts of mineral additions. This is necessary because the production of HPC/UHPC uses mineral additions with greater reactivity than those usually considered in the production of commercial cement, such as blast furnace slag and fly ash [13]. Different denominations are used for these special OPCs. According to the main international standards, these cement are known as CP-I. CP-V-ARI cement according to the Brazilian standards NBR 16697 [47]. CEM I cement according to the European standard EN 197-1 [48]. Type I cement according to the American ASTM C150 [49]. All these cement have in common their high clinker content, usually above 90% or 95%, and also high fineness. However, it should be noted that CP-I cement is not a commercial product, which is why research-based on NBR 16697 [47] focuses on the use of CP-V-ARI.

In particular, the use of one of these types of OPCs, the Brazilian CP-V-ARI, is cited as an example. Silva et al. (2020) [50] studied the effect of high temperatures on the mechanical performance of HPC/UHPC containing recycled aggregates and CP-V-ARI. This cement had a compressive strength at 7 days of 37.4 MPa and at 28 days of 43.7 MPa. The reference concrete compositions presented a compressive strength of 65.2 MPa, proving that it is an HPC. Roberti et al. (2021) [13] studied the autogenous shrinkage effects and the fresh and hardened state properties of an HPC. They used cement CP-V-ARI, with a strength of 38 MPa at 7 days and 45.8 MPa at 28 days. The evaluated compositions showed compressive strength from 68.3 to 84.3 MPa. Viana et al. (2020) [51] used CP-V-ARI to evaluate the influence of the incorporation of carbon nanotubes in HPC, obtaining compressive strength for concrete around 80 MPa at 28 days of cure. De Matos et al. (2020) [52] used CP-V-ARI to produce UHP cement pastes with 28-day strength of around 130 MPa. Pilar et al. (2021) [53] studied the rheological behavior of HPC/UHP using cement type CP-V-ARI, however, the authors did not report the results of compressive strength since the focus of the work was to evaluate the properties of the fresh state.

It is also important to mention other investigations on special processing and aggregates. Sohail et al. (2021) [1] studied the durability characteristics of HPC and obtained concrete with compressive strength of a little less than 100 MPa at 28 days. Storm et al. (2021) [54] evaluated the ways in which different fibers were pulled out in high-performance concrete. Li and Zhang (2021) [55] studied the thermal stresses in UHPC containing polypropylene and steel fibers, obtaining a compressive strength of 141.5 MPa. Liu et al. (2021) [56] evaluated the application of steel slag as a complementary material in UHPC, obtaining strength between 120 to 150 MPa. Suescum-Morales et al. (2021) [57] evaluated the effect of temperatures on high-performance concrete performing a microstructural analysis. Rashid et al. (2020) [58] evaluated the effects of using magnetite sand in UHPC, obtaining compressive strength of 134 MPa at 28 days. Olawuui et al. (2021) [59] evaluated the development of initial and long-term strength of HPC containing polymer incorporation, obtaining a strength of approximately 80 MPa at 28 days. Zhang et al. (2021) [60] valuated the fragmentation strength and mechanical properties of UHPC at high temperatures, obtaining strength around 130 MPa.

On the use of type I OPC, it is worth mentioning the following works. Choi et al. (2021) [61] evaluated the effect of TiO_2_ as a filler in concrete, obtaining compressive strength above 150 MPa at 28 days, typical of UHPC. Khan et al. (2020) [62] developed an ultra-high performance concrete for shielding from nuclear radiation, with a strength greater than 160 MPa. Kim et al. (2021) [63] used an OPC with a strength of 42.5 MPa to evaluate the benefits of curved steel fibers in the pullout strength of HPC. Yoo et al. (2021) [64] evaluated the effect of glass powder on the mechanical properties of UHPC, obtaining compressive strength greater than 200 MPa. Manigandan et al. (2021) [26] evaluated the use of treated banana fibers in the compressive strength of HPC, obtaining a value of approximately 52 MPa. Other researchers such as Yoo et al. (2021) [65], Kareem et al. (2021) [66], and Bae and Pyo (2020) [67] also used type I cement in their research with HPC and UHPC.

Regarding the chemical composition of the OPCs considered for the production of HPC and UHPC, it is observed that there are no major differences between the OPCs used for the production of CC, as shown in Table 2. The typical OPC base is essentially the same: between 60 to 72% of CaO, between 14 and 22% of SiO_2_, resulting in approximately 80% of the cement composed of CaO + SiO_2_. One can note that virtually all the cement presented in Table 2 had appreciable values (up to ~4%) of loss on ignition (associated with the CO_2_ thermal decomposition) and/or MgO. This can be explained by the presence of carbonaceous fillers, e.g., calcite (CaCO_3_) and/or dolomite (CaMg(CO_3_)_2_), or periclase (MgO). However, from a mineralogical point of view, it is relevant that the cement used for the production of HPC must present higher amounts of alite (C_3_S) and belite (C_2_S), which are the constituents of the OPC responsible for the formation of C-S-H, the main strength product of concrete [30]. Therefore, the CaO content must be analyzed coupled with the loss of ignition value, since this element can be present either as calcium silicate (i.e., C_3_S and C_2_S) or calcium carbonate filler. The content of C_3_A must be reduced because this mineral is incompatible with the conventional water-reducing chemical additives used since it mainly forms ettringite which adsorbs the additive molecules and tends to increase the additive content required to reach proper flowability [68,69]. This is the reason why cement must have the lowest possible amounts of Fe_2_O_3_ and Al_2_O_3_.

Regarding other important properties of OPCs, it is noteworthy that the density of OPC for HPC and UHPC and for conventional applications is the same, around 3.10 to 3.15 g/cm^3^. This is due to the low content of additions, since silica fume and fly ash, for example, have a specific mass around 2.2 to 2.4 g/cm^3^. The fineness, in general, is greater, due to the use of high initial strength OPCs, which are generally thinner, and due to the low amount of water used, generally less than necessary for the hydration reaction. So, greater fineness helps in a considerable degree of hydration, which is why OPCs need to be thinner. The same happens with the percentage of material retained in the #200 sieve, considered an important parameter for post-production OPC grinding control. This information is summarized in Table 3, where it is observed that the fineness of the OPC varies in values above 3500 cm^2^/g by the Blaine method. In general, the OPC used for conventional concrete presents a fineness close to 3000 cm^2^/g. Regarding the percentage retained in the #200 mesh sieve, it is observed that the maximum value observed was 2%, much lower than other types of OPC used for CC production, such as CP-II, CP-III, CP-cement V, cement type II and III and CEM II. This is necessary because the larger specific area allows these cement to react faster than those used for conventional applications.

Based on this, it is possible to establish that the OPC used in HPC and UHPC must be chemically rich in calcium and silica, mineralogically rich in C_3_S and C_2_S, with the least number of mineral additions possible, aiming at the incorporation of more reactive pozzolans than those used in OPC commercial.

### 2.2. Mineral Additions

Mineral additions are supplementary cementitious materials used together with OPC, with the aim of providing differentiated technological performance to cementitious products. Initially emerged as a need to improve some properties of concrete, it now contributes considerably to environmental aspects [75,76]. The need to replace Portland clinker with low-impact binders is today a common desire, but without losing the properties provided by these binders [77]. This is because ordinary cement is the world’s second most used material, second only to water. In addition, OPC production releases great amounts of CO_2_ mainly due to limestone decarbonation, necessary for clinker production [78]. Another important factor is the high energy demand required to reach a burning temperature of around 1500 °C. As a consequence, some authors point out that for every 1 ton of clinker produced, about 0.8–1.0 tons of CO_2_ are emitted into the atmosphere [76,79,80]. A portion of this emission is mitigated through CO_2_ uptake by mortar and concrete carbonation [78,81,82]; however, this topic will not be further addressed in this paper.

As a result, some researchers have proposed the replacement of clinker by agro-industrial residues or by-products. This is the case of blast furnace slag, fly ash, silica fume, and other pozzolans such as agricultural ashes. It was observed a reduction in production costs, due to the reduction in the consumption of clinker, which generally has a higher cost than aggregates. For example, Li and Jiang (2020) [83] observed a 21% cost reduction when used 60% slag and 10% limestone in OPC replacement for the same concrete strength class. Zhang et al. (2021) [84] demonstrated that concrete mix design can be optimized simultaneously for environmental, economic, and mechanical objectives with silica fume incorporation. In addition, there is a contribution to sustainable development, and the achievement of concrete with greater mechanical strength, especially with the use of pozzolanic materials [85,86,87].

Mineral additions can be classified according to their reaction with the clinker as inert. This, in general, only contributes to a physical filling effect improving the packaging. For example, this happens with the application of limestone filler as well as in cement, such as blast furnace slag, and in pozzolanic products, such as metakaolin, fly ash, agro-industrial ash, and silica fume [85,88,89]. Cementing additions are materials, usually based on calcium, that present an accelerated reaction capacity in the presence of alkaline media, as is the case of Ca(OH)_2_ of portlandite present in OPC [90,91]. They can also be activated with the use of other alkaline hydroxides, such as NaOH and KOH, through alkaline activation, dispensing in this case, the presence of OPC [92,93]. This type of reaction will be described in Section 4.

However, the additions with the greatest potential for the production of HPC/UHPC are the pozzolanic ones that in addition to the physical packing effect, described above, have a chemical effect. Pozzolans are materials based on silica or silica and alumina which, isolated, do not present any binding power, but when finely ground, and in the presence of clinker and water, develop binding powers [94,95]. This happens through the so-called pozzolanic reaction. It is observed that portlandite, formed through the hydration of the silicates present in the OPC, reacts with the amorphous silica and alumina present in the pozzolan and forms C-S-H and C-A-H. As aforementioned, the compound responsible for the strength of hardened OPC is C-S-H. Thus, the conversion of CH to C-S-H provided by pozzolans contributes significantly to the strength of the formed product [94,96].

It is observed that mineral additions have certain requirements for their efficiency in clinker replacement. From a physical point of view, they need to present a high specific surface, measured by the Blaine fineness, for example, to increase the contact area of mineral additions with OPC [96]. Mineralogically, these materials need to be predominantly amorphous, which means that they present structural disorders. Otherwise, the additions are not reactive, as crystalline materials have an organized structure and hardly change their structure under normal conditions of temperature and pressure due to hydration reactions.

From a chemical point of view, there is a wide range of mineral additions used. According to the bibliography [97,98,99], there are reports of the use of materials rich in calcium, such as furnace slag, limestone filer, and class F ash as well as materials rich in silica or aluminosilicates, such as fly ash, silica fume, and metakaolin, which make up the class of pozzolans.

One of the most used additions is fly ash, a by-product of thermoelectric plants that burn coal to produce energy. Ash is trapped in the combustion gas exhaust system and, when pulverized, it acquires pozzolanic characteristics [95,100]. Another great advantage of fly ash is the spherical shape of the material, which can promote a rolling effect between grains and improve workability, providing a reduction in the amount of water and consequently contributing to mechanical strength. This material is mineralogically amorphous and has a chemical composition of approximately 45–60% SiO_2_, 30–32% Al_2_O_3_, in addition to Fe_2_O_3_ and CaO in variable but detectable contents [101,102,103]. As thermoelectric plants are the main energy sources in the world, with the exception of some countries such as Brazil, the availability of fly ash is high, which justifies the wide application of the material as pozzolans [103].

In the research by Mohan et al. (2021) [101], in which the authors used two different types of fly ash as additions for HPC/UHPC, the proportion of additions used was approximately 50% of the OPC mass. The authors obtained a concrete with 69 MPa compressive strength at 28 days. The two fly ashes used, named 1 (59.32% SiO_2_, 29.95% Al_2_O_3_, 4.32% Fe_2_O_3_, 1.28% CaO) and 2 (60.56% SiO_2_, 32.67% Al_2_O_3_, 4.44% Fe_2_O_3_, 1.41% CaO), presented a fineness by the Blaine method of 5636 and 6210 cm^2^/g, respectively. Comparing with the OPC used by the authors, with a fineness of 3300 cm^2^/g, it is observed that their additions were finer than this binder.

**Figure 1:**
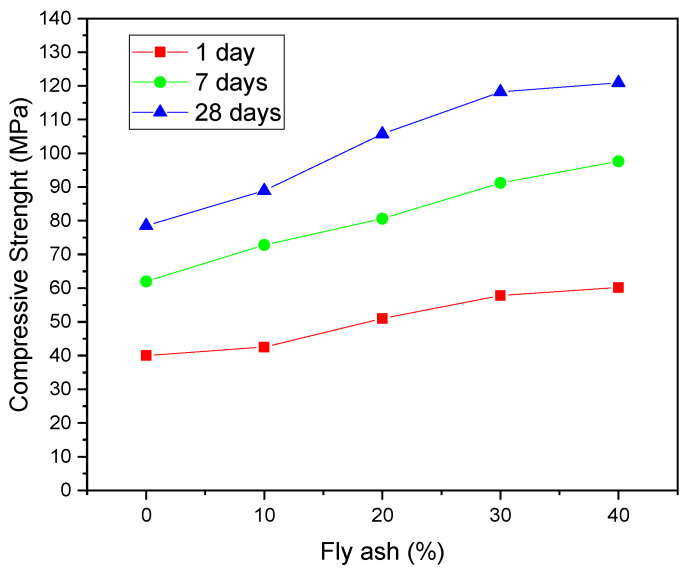
Influence of fly ash content on the compressive strength of ultrahigh performance concrete [105].

Other studies with the application of HPC/UHPC that used fly ash are highlighted below, to justify that this material is one of the most used additions in high-performance concrete: Sujay et al. (2020) [104] studied the effect of the application of steel fibers in high-performance concrete containing mineral addition of fly ash replacing 15% of the OPC mass, obtaining compressive strength at 28 days of approximately 55 MPa. Bahedh and Jaafar (2018) [105] studied the application of fly ash (69.41% SiO_2_, 28.20% Al_2_O_3_, 5.30% Fe_2_O_3_, 6.47% CaO) to replace OPC in percentages of 0–40% for the production of UHPC by molding in an autoclave, with the application of pressure in the production stage of the specimens. The authors observed that the use of 40% ash allowed to obtain a compressive strength at 28 days of 120 MPa, while the reference composition presented a strength of 80 MPa at that same aging period. This information is illustrated in Figure 1.

Zhang et al. (2020) [106] studied the effects of replacing 25% OPC by fly ash (50.35% SiO_2_, 29.65% Al_2_O_3_, 6.61% Fe_2_O_3_, 5.85% CaO) in HPC subjected to situations of high temperatures. The authors obtained a compressive strength at 28 days of approximately 55 MPa for the composition containing ash, while the reference composition showed a strength of less than 50 MPa, not being characterized as HPC. At all ages evaluated, the compositions containing fly ash showed a better mechanical performance, which is directly related to the pozzolanic effect.

Choudhary et al. (2021) [107] evaluated the effect of various mineral additions on the abrasion and mechanical strength properties of high-performance concrete. Although the best results were obtained with the use of silica fume (95.58% SiO_2_, 0.71% Al_2_O_3_, 0.81% Fe_2_O_3_, 0.90% CaO), which will be discussed below, the results obtained with fly ash (58.19% SiO_2_, 26.93% Al_2_O_3_, 4.27% Fe_2_O_3_, 0.90% CaO) were also positive. For example, obtaining compressive strength at 28 days of 55 MPa. One of the reasons that explain the better performance of silica fume is the fineness of the material, 9550 cm^2^/g by the Blaine method, while fly ash showed 3530 and cement 2860 cm^2^/g. Another explanation is the chemical composition since silica fume has a higher SiO_2_ content than fly ash.

As already emphasized, silica fume is another pozzolanic mineral additive used in HPC/UHPC, with mechanical properties superior to those obtained by using fly ash. In the research by Choudhary et al. (2021) [107], cited in the previous paragraph, the compositions with silica fume obtained compressive strength at 28 days of 75 MPa, while the same compositions containing fly ash obtained 55 MPa. Some relevant information helps to explain this difference in behavior.

Silica fume is a by-product obtained in the production process of plain silicon or in silicon iron alloys. The process is carried out in large metal furnaces, by reducing the quartz in the presence of coal, or iron in the case of alloys, at very high temperatures, around 2000 °C. During the heating step, silicon monoxide (SiO) is eliminated as a gas, oxidizing and condensing into extremely small spherical particles of amorphous silica (SiO_2_) [108,109]. In terms of chemical composition, silicas fume have a SiO_2_ content above 95%, another characteristic that contributes to their pozzolanic effect [102,107].

This produced SiO_2_ material is trapped in the furnace exhaust gas filtration systems, being removed and used as a pozzolan. The average diameter of the material is 0.1 mm, about 100 times smaller than the average diameter of the OPC particles, presenting a specific surface by Blaine fineness in the order of 9000 to 10,000 cm^2^/g [109,110]. This contributes to the greater reactivity of this addition, in addition to contributing to granular packing and helping in pozzolanic reactions in regions that other conventional pozzolans cannot, such as at the paste-aggregate interface. However, it should be noted that silica fume, unlike fly ash, has a higher commercial value, as the production numbers of the silicon industry are much lower than the production of fly ash in thermoelectric plants [110,111].

Some researches that studied the application of silica fume are highlighted below. Wu et al. (2019) [112] studied the changes in rheological and mechanical properties with the use of silica fume (95.2% SiO_2_) in ultra-high performance concrete. The authors obtained strength of 120 MPa at 28 days of cure, with the use of 20% silica fume (94.8% SiO_2_) to replace OPC. Smarzewski et al. (2019) [113] used silica fume to evaluate the mechanical properties of UHPC, obtaining a strength of 95 MPa for the reference composition and 110 MPa for the composition containing 20% of the additive. Pedro et al. (2017) [114] and Pedro et al. (2018) [115] evaluated the mechanical properties and durability of HPC produced with recycled aggregate and silica fume (94.2% SiO_2_). The authors obtained compressive strength of 76.70 MPa after 28 days with the compositions containing silica fume in the first study and observed that the performance in the durability tests was superior for the compositions containing 20% silica fume, in all evaluated conditions (strength to carbonation, strength to chloride attack, water absorption by immersion, and by capillarity) in the second study.

**Figure 2:**
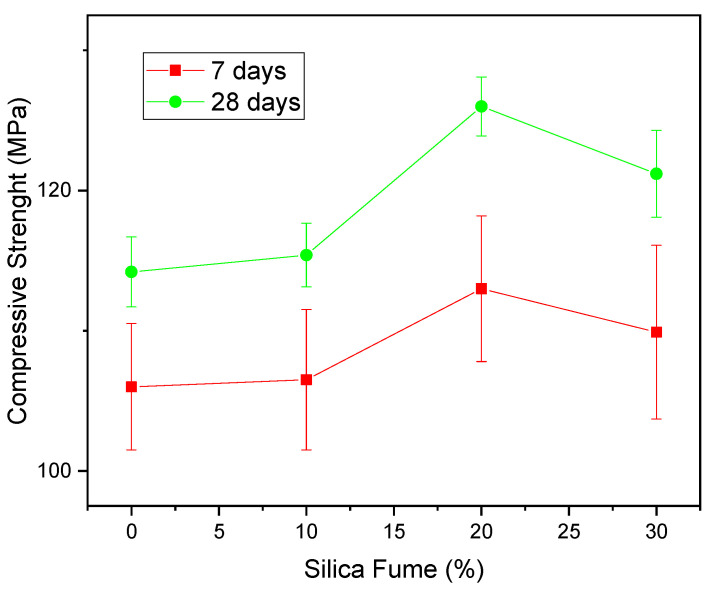
Compressive strength of Ultra-High Performance (UHPC) containing silica fume [116].

Chen et al. (2018) [116] studied the application of fly ash to replace OPC in percentages of 0–30% for the production of UHPC by molding in an autoclave, with an application of pressure in the production stage of the specimens. The authors observed that the use of 20% ash allowed to obtain a compressive strength at 28 days of 125 MPa, while the reference composition presented a strength of 105 MPa at the same age, as shown in Figure 2. This change in strength was attributed by the authors to the greater formation of C-S-H, which is directly attributed to the pozzolanic reaction promoted by the material, as well as to the likely improvement in granular packing, related to the physical effect. In addition to the aforementioned research, several other studies used silica fume as a mineral addition [68,117,118,119,120,121].

Other incorporations are also used in the production of HPC and UHPC, although in much smaller proportions than fly ash and silica fume. Several countries, such as China, Japan, Brazil, the USA, India, and Germany, have a great potential for using blast furnace slag, obtained through the steel industries, a strong industrial sector in these countries. Blast furnace slag is generated as a by-product of the production of pig iron with a high calcium content [122,123]. The same can be highlighted in the ceramic industry, a strong sector in countries such as China, Brazil, Italy, and Spain, responsible for the production of ceramic waste and metakaolin, two pozzolans with a high content of silica and alumina [124,125,126]. Several countries, such as China, the USA, Argentina, and Brazil, also present the possibility of using agro-industrial ash, such as rice husk ash and sugarcane bagasse ash, creating alternatives for the application of renewable forms of pozzolans [127,128,129].

On the use of slag, the following works stand out. Shen et al. (2020) [130] and Shen et al. (2020) [131] studied the use of blast furnace slag in HPC obtaining a strength of 52 and 66.9 MPa, respectively. Cheah et al. (2019) [132] and Ma et al. (2018) [133] studied the use of blast furnace slag together with fly ash for the production of high-performance concrete, obtaining strength at 28 days of approximately 50 MPa. On the application of ceramic waste, it is worth mentioning the following research works. Kannan et al. (2017) [134] obtained a concrete of 51.5 MPa at 28 days with the replacement of 10% OPC by ceramic waste. Xu et al. (2021) [135] obtained a UHPC of 120 MPa at 28 days using 15% ceramic waste as pozzolan replacing OPC. Salami et al. (2020) [136] studied the application of metakaolin as a mineral additive in HPC obtaining a compressive strength of 60 MPa. Song et al. (2019) [137], Shehab et al. (2017) [138], and Tafraoui et al. (2016) [139] studied the use of metakaolin as an additive in UHPC containing different types of fibers. All authors obtained compressive strength at 28 days above 100 MPa.

Regarding the use of agro-industrial ash, the research by Le and Ludwing (2016) [140] stands out, which evaluated the use of fly ash, silica fume, and rice husk ash together for the production of UHPC, obtaining a strength of 110 MPa at 28 days. Le et al. (2015) [141] evaluated the durability of HPC containing rice husk ash as a pozzolan. Shaaban et al. (2021) [142] evaluated the rheological and hardened state properties of HPC containing this same additive, obtaining a strength of 60 MPa at 28 days. Finally, the research by Gar et al. (2017) [143] studied the use of sugarcane bagasse ash as pozzolan in HPC, obtaining strength at 28 days of 52 MPa.

### 2.3. Aggregates

Aggregates are defined as inert, granular materials without defined shape and volume, used in concrete for economic and technological reasons [144]. They are classified according to their origin as artificial (or industrialized), natural, or recycled. Crushed stones are cited as an example of artificial aggregates. Other examples are natural sand washed from the river and construction and demolition waste used after recycled [145,146].

As for their own weight, aggregates are classified as light, such as expanded clay, conventional, such as crushed stone, or heavy, such as hematite aggregates [144,147]. From a granulometric point of view, they are classified into coarse, those whose grains pass through the 152 mm opening sieve and are retained in the 4.75 mm sieve, and in giblets, those whose grains pass through the 4.75 mm opening sieve and remain retained in the 0.075 mm aperture sieve [145,148]. The particle size distribution of the materials plays a major role in the fresh and hardened performance of HPC and UHPC. While the aggregates correspond to the macroscale components, the binder materials (i.e., OPC and mineral additions) correspond to the microscale fraction of concrete. In addition, the very small silica fume particles can improve the particle packing of the binder fraction, leading to higher compactness due to physical effects besides the pozzolanic contribution [149]. By optimizing the particle size distribution and mix proportions, one can achieve maximum particle packing, therefore improving the fresh and hardened properties of concrete. This strategy has been widely used for HPC and UHPC design over the last years [150,151,152].

Dealing specifically with the aggregates used in HPC, it is observed that there is no specificity regarding the use of these materials when compared to conventional concrete. However, care must be taken with regard to the particle size of the materials, in order to find the packing of all aggregates, following a continuous distribution, which presents the smallest possible void volume. This characteristic is even more important in the production of UHPC, which usually do not have coarse aggregates in their composition, due to the possible presence of micro-cracks caused by crushing or because the strength of the coarse aggregate is usually inferior to that of the cement matrix, making the aggregate the fragile point of the material. Owing to the above-described reasons, it is common for authors to use more than one type of fine aggregate or different combinations of fine and large aggregate to obtain the best packing. Arunothayan et al. (2021) [70], for example, uses three different types of sand as fine aggregate for HPC production used in 3D printing applications. With the different combinations proposed, the authors obtained compressive strength ranging from 110.1 to 152.5 MPa.

In addition to aspects related to packaging, some physical parameters need to be analyzed. Regarding the coarse aggregate used for HPC, the following points are worth mentioning. The content of fines, passing through the 75 mm sieve, should be limited to 1%, as this material is generally attributed to silt and clay particles, which can increase the aggregate water absorption. The D/d shape ratio, which relates to the largest size and smallest aggregate size, should be limited to 3 to avoid anisotropy in concrete. Water absorption should also be limited to 7%, as should abrasion wear strength measured by the Los Angeles method, which should be restricted to 50%.

**Figure 3:**
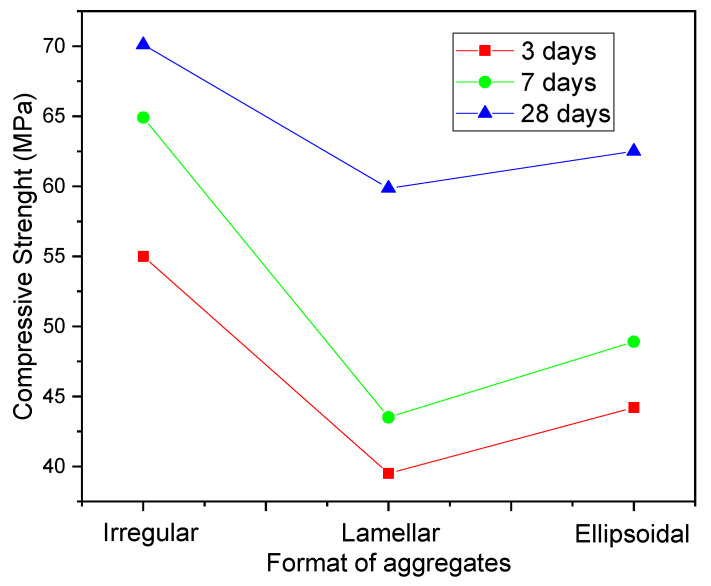
Compressive strength results as a function of aggregate shape [153].

On the D/d ratio, Zhao et al. (2021) [153] evaluated the influence of three coarse aggregate geometry on the mechanical properties of HPC. The authors used lamellar, irregular, and rounded aggregates. The strength results obtained by the authors are highlighted in Figure 3, where it is possible to observe that the best results are obtained with irregular aggregates. This is attributed by the authors to greater adherence between the cementitious paste phases and the aggregates, which improves the behavior of HPC.

Regarding the physical characteristics of small aggregates, some information is pertinent. For example, the number of fines is also limited, but to a total of 3%. The limit water absorption is 7%, while the swelling coefficient must be as low as possible, to avoid excessive volume increase. There are still some recommendations related to concrete durability problems, which apply to both coarse and fine aggregates. The content of chlorides and sulfates, for example, should be limited to 0.1% of the chemical composition of the aggregates. This is necessary to avoid the occurrence of oxidation in the concrete reinforcement and to avoid the formation of late ettringite in the concrete, causing an expansive reaction that destabilizes the material volume and generates internal stresses [154]. Problems related to alkali-aggregate reaction (AAR) must also be verified through the mortar bar test. The maximum expansion allowed in this test is 0.05% after 3 months and 0.10% after 6 months. In AAR, a gel is formed that absorbs water and tends to increase the volume of the concrete, which can generate cracking and disaggregation of the aggregate paste [148,155].

Analyzing the researches published with HPC and UHPC, it is observed that the authors traditionally use quartz sand washed from the river [1,3,13,70] or quartz sand from dunes [62,156] as the main fine aggregate. As a coarse aggregate, it is common to use crushed stones [25] and gravel [157]. The stones used are limestone [114,115,158] and diabase [159], mainly.

**Figure 4:**
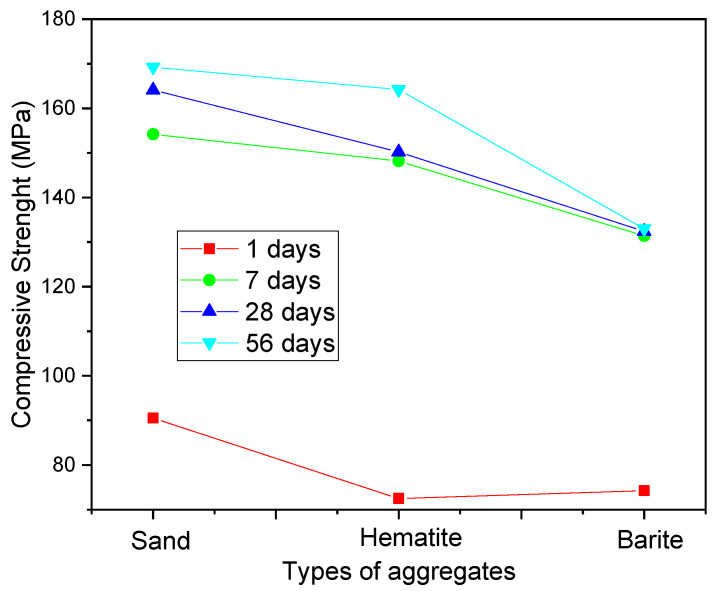
Compressive strength due to different types of aggregate [147].

There are also researches using heavy aggregates such as barite, magnetite, and hematite for HPC in nuclear protection applications [147,160,161]. Figure 4, for example, shows the compressive strength results obtained using 3 types of fine aggregate: silica sand (97.32% SiO_2_), barite (58.69% BaO), and hematite (71.71% Fe_2_O_3_). The silica sand used has a specific mass of 2.7 g/cm^3^, while the barite has 3.0 to 4.4 g/cm^3^. Although the compressive strength results are better for the composition with silica sand, the radiation absorption values of cobalt and cesium were much higher for heavy concrete manufactured with barite, justifying its use in this type of application.

There are also researches that use light aggregates, mainly expanded clay, for the production of HPC. Angelin et al. (2020) [162] evaluated the packing of lightweight concrete containing expanded clay and rubber as aggregates, obtaining strength at 28 days of 58.5 MPa. Lu et al. (2021) [163] also obtained compressive strength results compatible with HPC using expanded clay aggregates. However, Garcia et al. (2021) [164] reported that the use of expanded clay in high-performance concretes is problematic, due to defects arising from the calcination of these aggregates. As a result, it is usual to increase OPC consumption and use a high number of additives, in addition to reducing the w/c factor, which is only possible using considerable amounts of plasticizer additive. This makes concrete too expensive and is therefore not recommended.

Research that use recycled aggregates for the production of HPC and UHPC are also mentioned. These researches use aggregate from concrete waste [114,115] and ceramic waste [157,165]. Using these residues, which present the particle size curve within the normative limits of the ASTM C33 standard, the authors obtained a compression strength after 28 days of curing of 77.3 MPa, proving the feasibility of using recycled aggregates, as long as they meet the stipulated granulometry parameters.

Based on this, it is observed that the choice of aggregates for application in HPC and UHPC should be more carefully selected for application in CC, due to the need to obtain greater packaging. It is possible to use not only coarser and finer but also conventional and heavy aggregates, as well as lighter and recycled aggregates for the production of HPC and UHPC.

### 2.4. Chemical Additives

Chemical additives are materials used in the production of concrete aiming to improve properties of interest. They are generally used in quantities of up to 5% of the OPC mass [166]. The main types of chemical additives are air-incorporated additives, tack modifying additives, water-reducing additives, and shrinkage-mitigating additives, especially used in UHPC. As the name suggests, air-incorporated additives add air bubbles to concrete voids, improving the plasticity and workability of the material, but potentially compromising compressive strength. They are used in situations where concrete is subjected to ice and thaw, acting as a kind of reservoir where water can migrate, since when freezing the water expands, which could cause the concrete to crack [166,167,168].

Most used shrinkage mitigating additives are amphyphylic molecules, which have a hydrophilic end and a hydrophobic end. When interacting with the water present in the hydration of OPC, or any other polar solvent, the molecules of these additives are mainly absorbed in the liquid-vapor interface by electrostatic repulsions due to the interaction of hydrogen bonds [169,170]. In this way, there is a reduction in the surface tension of the water in the pores of the concrete, allowing a reduction of up to 50% in concrete shrinkage. The reduction is possible because shrinkage-mitigating additives continue to act in the pore system even with hardened concrete, reducing the effects of water surface tension that contribute to drying shrinkage [170,171]. The main examples of such additives are propylene glycol, general glycol ethers, and polyethylene glycol [169].

Set-modifying additives serve to delay or accelerate the setting time and hardening time of concrete. They do not significantly change the final strength of concrete, however, they do change the strength at early ages [172]. The water-reducing additives, on the other hand, serve to reduce the w/c ratio without losing the workability of the concrete. They are the main additives used in the production of HPC and UHPC [173,174]. In addition to these additives, there are polyfunctionals that have two or functions simultaneously, generally modifying not only the set but reducing the amount of water.

As mentioned, water-reducing additives are the most used for the production of HPC and UHPC. These additives are subdivided into three generations. Depending on the amount of water they reduce in the concrete, these are: (i) 1st generation of superplasticizers that reduce from 6 to 12% of water; (ii) 2nd generation of superplasticizers that reduce 12 to 20% of water; and (iii) 3rd generation of superplasticizers that can reduce water above 20%, reaching a reduction of up to 45% of the mixing water [174,175]. In HPC and UHPC, 3rd generation superplasticizers are preferably used. The importance of water reduction is related to Abrams’ Law, which indicates that the lower the w/c ratio, the greater the mechanical strength, using the same material parameters [176]. Logically, the reduction of the w/c factor impairs the workability of the concrete, which is why chemical additives are used.

The water reduction promoted by 1st and 2nd generations of superplasticizer reducers can be explained through the electrostatic dispersion effect. This effect occurs because the additive involves a system of OPC particle charges of the same sign [177]. Due to the effect of electrostatic repulsion, the superplasticizer will disperse the cement particles, making less water necessary to reach a given workability [178,179].

The 3rd generation of superplasticizers works due to the steric effect or due to the combination of the electrostatic repulsion effect with the steric effect [180]. This effect, which occurs mainly in additives based on polycarboxylate (PCE), the main additive used in HPC and UHPC. PCE features a long main chair, with shorter branches and side chains, increasing floor space in an OPC particulate system, resulting in much greater water reduction than 1st and 2nd generation plasticizers [181,182,183].

As examples of some additives used in HPC, the following works stand out. Ibragimov and Fediuk (2019) [184] evaluated the influence of different types of superplasticizers on the mechanical properties of concrete. The authors used five different superplasticizer additives: the first is a copolymer based on polyoxyethylene derived from unsaturated carboxylic acids (1st generation); the second is based on sodium salts of polymethylene naphthalenesulfonic acids (2nd generation); the third is a polyfunctional consisting of naphthalenesulfonate and an organic accelerator; the fourth additive used is a superplasticizer based on polyoxyethylene derivatives of polymethacrylic acid (PAA); finally a copolymer based on polyether carboxylates (PCE). The strength results obtained by the authors are shown in Table 4. It is observed that the additives that contributed the most to the compressive strength were the PAA and PCE, which is why they are the most used in the literature.

Benaicha et al. (2019) [185] analyzed the effects of superplasticizer additives on the rheological and strength properties of HPC. They used PCE-type superplasticizers in different percentages, obtaining a compression strength of 73.49 MPa at 28 days with the use of 0.3% of the superplasticizers. Cheah et al. (2020) [186] evaluated the changes in the mechanical and microstructural properties of HPC produced with PCE-type superplasticizers containing a ternary mixture of OPC, blast furnace slag, and silica fume. The authors obtained results compatible with the behavior of HPC, obtaining compressive strength at 28 days of around 80 MPa. Cheah et al. (2019) [132] evaluated the performance of HPC containing fly ash, blast furnace slag, and PCE-type superplasticizers. The compression results obtained were consistent with HPC applications. Finally, the work by Guan et al. (2021) [179] reported the durability effects of HPC sulfates produced with PAA-type superplasticizers. The authors did not perform mechanical tests but emphasize that the applied concrete presents behavior for high-performance applications.

Thus, based on what has been presented, it is observed that the use of superplasticizers is essential to obtain an HPC and UHPC with an adequate behavior. The most used additives are PCE, 3rd generation of superplasticizers that work by the combined principle of electrostatic repulsion with steric effect.

## 3. HPC and UHPC Containing Fibers: Composite Materials

High-performance fiber reinforced concrete (HPRFC) or ultra-high-performance fiber reinforced concrete (UHPFRC), emerged as a need to improve ductility and tensile strength properties in HPC and UHPC [25,70]. Research with this type of material is relatively recent, starting from the 1990s, about 30 years ago [187]. The researchers observed that, although the mechanical behavior related to compression was increasing, the same did not happen with tension, which compromised the concept of high performance [188].

In this context, a new perspective of HPC and UHPC emerged: using the concepts of composites applied to concrete materials. Composites are materials that use two different components to obtain a material with superior properties, these two components are the matrix phase, the majority phase, responsible for involving and protecting the second phase, which is the reinforcement, dispersed throughout the matrix. The matrix of a composite can be metallic, polymeric, or ceramic. In the case of high-performance concrete, the matrix used is cementitious, essentially composed of HPC and UHPC [13,188,189]. Despite the great potential of using fibers to reinforce HPC and UHPC, few standards were developed to date, such as the French standard [37].

This concrete, used as a matrix, has the same components highlighted in Section 2, that is, OPC, mineral additions, preferably pozzolanic of high fineness, such as silica fume and fly ash, low w/c, requiring the use of superplasticizer chemical additives, and fine and coarse aggregates following the same specification detailed in Section 2.2.

**Figure 5:**
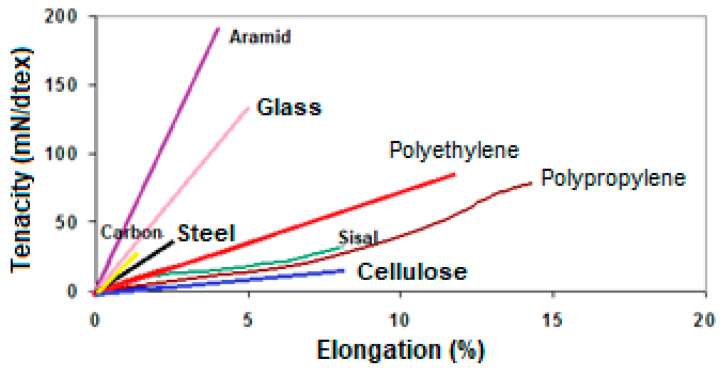
Stress x strain diagram of fibers used in HPC and UHPC [191,192].

The reinforcement phase of a composite can be in the form of filler particles or in the form of fibers. In the case of HPC and UHPC discussed in this section, the reinforcement phase is composed of synthetic fibers that can be metallic, polymeric, and ceramic, or natural fibers, of mineral, animal, and vegetable origin [12,13,190]. Figure 5 shows the behavior of tenacity and elongation at the break of the main fibers used for application in HPC and UHPC, while Table 5 shows the main properties of these fibers. The main advantages of applying fibers in HPC and UHPC are the high gain in mechanical strength, both in compression and in traction, and the increase in ductility. Disadvantages include the reduction in the workability of concrete, which can be solved using 3rd generation of superplasticizer reducers, and the higher cost of the material, especially with the use of synthetic fibers. These issues will be detailed in this section.

### 3.1. Steel Fibers

Through the consulted database, it is observed that the majority of research carried out with HPC and UHPC, mainly use steel fibers, produced through essentially ferrous metallic alloys, containing between 0.008 and 2.11% of carbon. These fibers present positive properties, such as high ductility and tensile strength, in addition to compatibility with concrete, which is typically observed in the use of steel bars in reinforced concrete [73,200,201,202]. However, they are prone to corrosion, which is why several authors have studied the effects of this pathology on the behavior of HPC. According to Shin and Yoo (2020) [203], Yoo et al. (2020) [204], Lv et al. (2021) [190], and Ngo et al. (2021) [205], corrosion reduces the strength of the cementitious composite and decreases its ductility to levels even worse than its behavior without reinforcement. Therefore, corrosion must be severely avoided.

**Figure 6:**
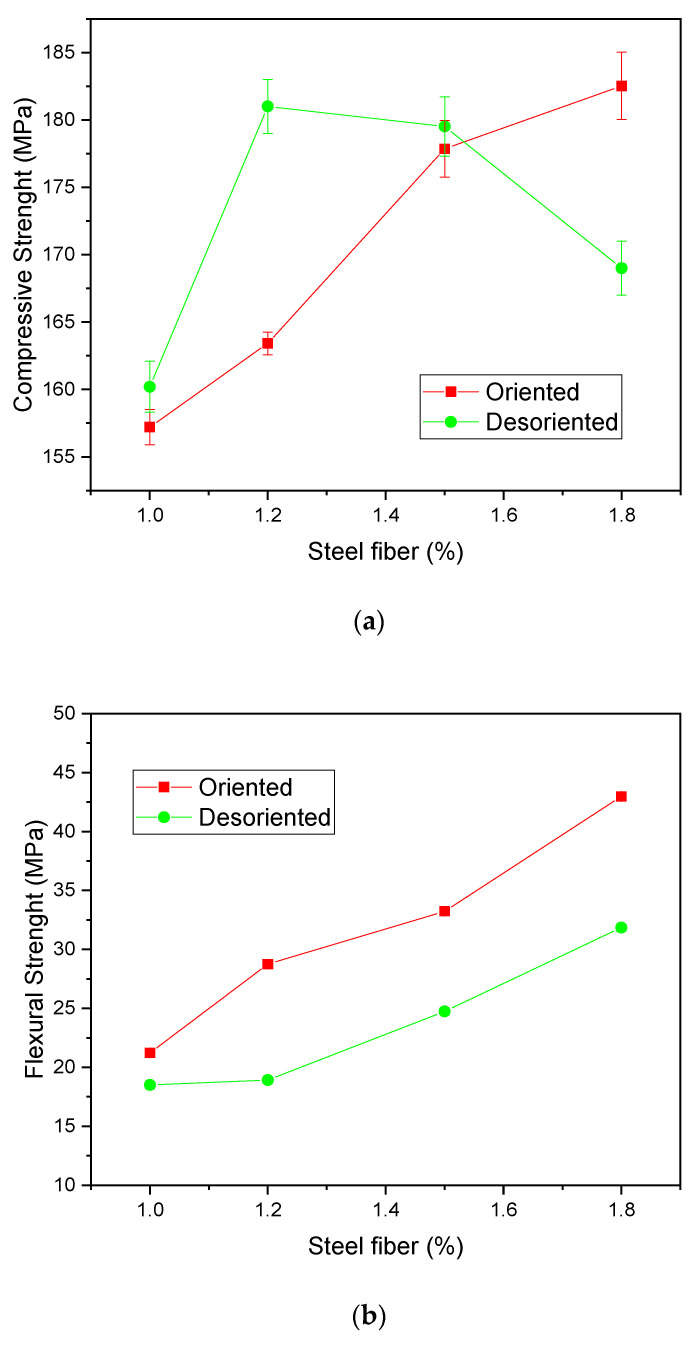
(**a**) Compressive strength, (**b**) flexural tensile strength fibers [206].

On the mechanical properties obtained by the application of steel fibers, Gou et al. (2021) [206], studied the mechanical properties of HPC containing different fractions of steel fibers in an orderly and disorderly manner in the cementitious matrix. The authors observed, as can be seen in Figure 6a, that the use of 1.0, 1.2, and 1.5% of disordered fibers presents compressive strength after 28 days of cure, superior to equivalent compositions with oriented fibers. However, this pattern of behavior is changed with the use of 1.8% of fibers because, as the fiber content is high, the workability of the cement mortar reduces, resulting in an agglomerate of steel fibers, which causes the formation of inter-defects in the concrete and, consequently, the loss of strength.

Regarding the flexural tensile strength at 28 days, Figure 6b, it was observed that the oriented fibers had better results, regardless of the amount of fiber evaluated. This is attributed by the authors to the formation of stress transfer bridges and crack propagation. This effect is obtained in greater intensity when the fibers are properly oriented. That is also why the results improve considerably with the use of higher amounts of fiber, where the composition with 1.8% oriented showed a result of 40 MPa, for example.

Other important works using steel fibers in HPC are now cited. Ashkezari et al. (2020) [201] studied the experimental relationships between the volume fraction of steel fiber and the mechanical properties of HPC. Bao and Pyo (2020) [67] evaluated the mechanical behavior and electrical properties of high-performance concrete containing fiber incorporation for application in railway sleepers. Park et al. (2021) [207] evaluated and verified the orientation and distribution of steel fibers in high-performance concrete pillars using computerized microtomography. Kim et al. (2021) [63] and Kim et al. (2020) [208] studied the benefits of applying curved steel fibers with a radius of 10–50 mm on the properties of high-performance concretes. Dingqiang et al. (2021) [209] evaluated the influence of the use of straight and short (6 mm), straight and long (13 mm) and hook-end fibers on the mechanical properties of UHPC, performing experimental tests and computational analyses. The best results were obtained using 2% steel fibers with a hook, obtaining approximately 150 MPa compressive strength and 45 MPa flexural tensile strength at 28 days. In this way, it was concluded that the steel fiber is compatible with the cementitious matrix for UHPC applications.

### 3.2. Other Synthetic Fibers

Other synthetic fibers used for HPC and UHPC applications are carbon, glass, and polymeric materials such as polypropylene and polyethylene. Carbon fibers, composed of thousands of unified filaments, have the advantage of greater adhesion to the cement matrix due to their high specific area.

Relevant researches with the application of carbon fibers in HPC and UHPC may cite the following: Afzal and Khushnood (2021) [210] evaluated the influence of carbon fibers on the performance of UHPC exposed to high temperatures; Jung et al. (2020) [211] reported on the changes in the structural behavior of HPC containing carbon fibers; Liu et al. (2020) [197] measured the strength gain of HPC containing carbon fibers at early and intermediate ages; Zhou et al. (2020) [212] evaluated the behavior of HPC e UHPC containing several types of fiber, including carbon, exposed to high temperatures.

Glass fibers are produced using borosilicate glass (E-glass) or soda-lime-silica (A-glass), with the advantages of being able to apply thin panels and elements without the occurrence of corrosion. However, they can present durability problems, since the cementitious matrix is highly alkaline, which can degrade the fiber and promote the embrittlement of HPC and UHPC [12,64]. Some important works with this fiber are worth mentioning. Bilisk and Ozdemir (2021) [213] studied the three-dimensional configuration of glass fibers in cementitious composites composed of HPC and UHPC. Al-Khafaji et al. (2021) [214] evaluated the behavior of sustainable HPC with ecological materials and fiberglass application. Kumar et al. (2021) [215] studied the application of UHPC containing glass fiber, and other fibers, for structural reinforcement of concrete and reported corrosion problems. Ali et al. (2020) [216] investigated the behavior of beams subjected to bending of UHPC containing recycled aggregates and fiberglass.

**Figure 7:**
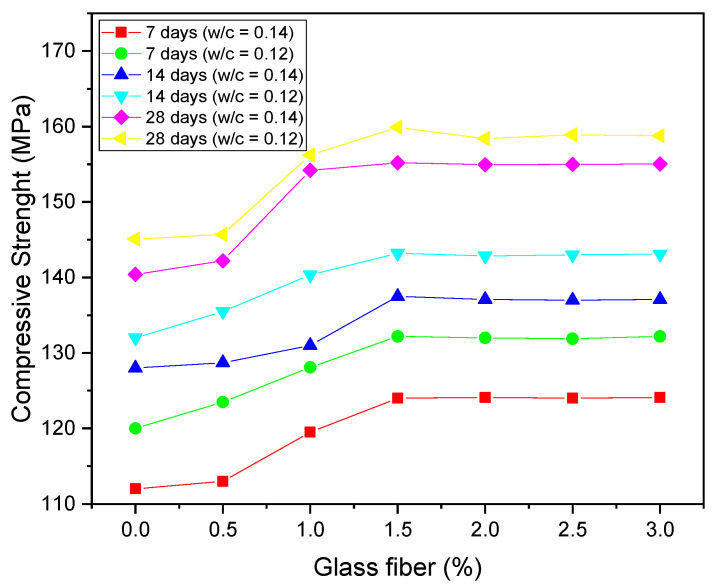
Compressive strength of HPC containing glass fibers [217].

Regarding the application of glass fibers, the work by Mohamed et al. (2021) [217] in which the authors applied glass fibers at various levels of UHPC with a w/c ratio of 0.12 and 0.14, varying the curing age in 7, 14, and 28 days, as shown in Figure 7, is worth mentioning. The authors observed that after using 1.5% fibers, the compressive strength results did not change. This happens because the composite has reached the fiber saturation point, which is the point at which the matrix is not wettable to absorb the fibers used. In concrete, this can cause a drop in strength or a loss of workability.

**Figure 8:**
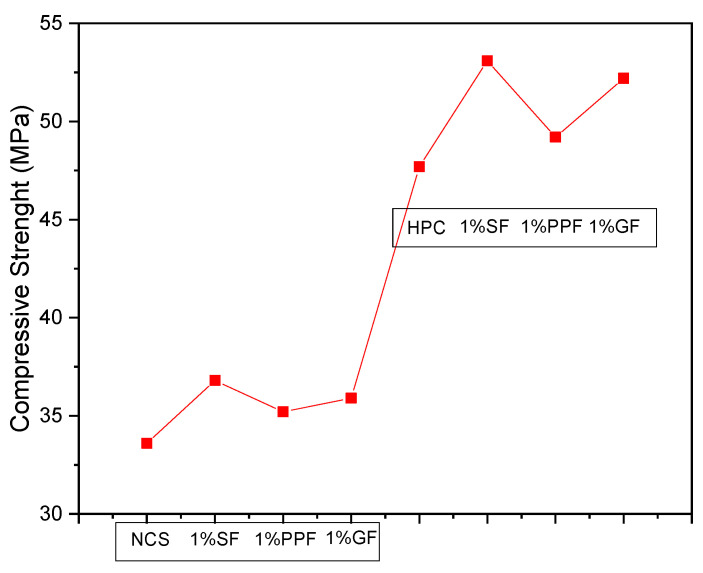
Compressive strength of HPC containing fibers: S (steel), G (glass), P (po polypropylene) and R (reference) [218].

The most used polymer fibers are polypropylene (PP) and polyethylene (PE). The main advantages are the low density of fibers when compared to others already mentioned in this review. As for disadvantages, the mechanical behavior is mentioned, which is inferior to steel, glass, and carbon fibers. Hussain et al. (2020) [218], for example, compared the mechanical behavior of CC and HPC containing 1% steel, glass, and PP fibers, as shown in Figure 8; as for HPC, steel fibers are the best performers, followed by glass. PP fibers have the worst results, even though they are superior to the reference concrete. However, as these fibers are lighter, their application should not be discarded, mainly because they help to reduce HPC and UHPC retraction.

Other important research that used polymeric fibers are now cited. Behafarnia and Behravan (2014) [219] evaluated the application of PP fibers in HPC to be used in water tunnels; Zhu et al. (2017) [220] evaluated the effect of the degree of aggregate saturation on the freeze-thaw strength of HPC e UHPC with light PP fibers; Li et al. (2018) [221] and Li and Zhang (2021) evaluated the influence of aggregate size and inclusion of PP and steel fibers on the hot permeability of UHPC at high temperature; Yan et al. (2021) [12] carried out experimental research on the increase of HPC ductility using basalt fiber, PP fiber, and glass fiber; Shen at al. (2020) [222] analyzed the effect of the length of PP fibers on the crack strength of high-performance concrete at an early age; Yoo and Kim (2019) [223] evaluated the influence of PE fibers on the mechanical and impact strength properties of HPC; and finally, He et al. (2017) [224] evaluated PE fiber coating mechanisms to improve the adhesion and mechanical properties of UHPC.

### 3.3. Natural Fibers

Natural fibers, such as fibers of mineral origin like basalt [12,212,225,226], and fibers of plant origin, like sisal [72,227], as well as banana [228,229], can also be used as reinforcement of HPC and UHPC. Their main advantages are the fact that they are renewable and eco-friendly resources. The main disadvantages are the great variability of properties and the possibility of degradation in an alkaline environment, especially in the case of fibers of vegetable origin that need previous treatments to be applied in cementitious matrices [72,227,230]. Another important advantage of natural fibers, especially vegetable fibers, is related to the fiber’s high adherence to the OPC matrix. The adhesion mechanisms of this type of fiber are related to the fibrous structure of the cellulose [72]. Some authors have carried out studies on the improvement of the adhesion properties of these fibers through treatments with alkaline materials, which attack the fiber surface, increasing the roughness and adhesion with the matrix [227,230].

## 4. HPC and UHPC Produced with Alkali-Activated Cement

Another type of HPC and UHPC that has emerged as a great potential for application in civil construction is based on alkali-activated cement [231]. This type of cement, unlike OPC, is composed of two materials: a powdery and amorphous component named as a precursor, and another liquid composed of an aqueous solution of a dilute alkali metal, named as an activator [232,233]. The types of precursors and activators will be presented in detail in the following sections; however, in summary, they must result in composites with similar characteristics to OPC-based HPC and UHPC: adequate flowability, high mechanical strength, and good durability.

From the point of view of aggregates, the same as conventional HPC and UHPC are used, namely: washed sand from rivers or crushed sand, as fine, as well as gravel or rolled pebbles, as coarse. Thus, the materials used as precursors and activators will be discussed in this topic, since these are the components that differentiate concretes produced from alkali-activated cement.

### 4.1. Precursors: Overview

Precursors, as highlighted, are very fine materials and are predominantly amorphous, which can have two basic chemical compositions: (i) rich in calcium or (ii) rich in aluminosilicates. To classify the material, the CaO/(SiO_2_ + Al_2_O_3_) mass ratio is calculated. If this ratio is greater than 1, the material is considered rich in calcium, as is the case with blast furnace slag and some types of fly ash. If the ratio is less than 1, the material is considered rich in aluminosilicates, giving rise to a subclass of alkali-activated materials known as geopolymers [234,235]. This is the case of metakaolin, ceramic waste, glass waste, and most types of fly ash.

The use of precursors, which totally replaces the application of OPC, presents as main benefits the possibility of applying residues and agro-industrial by-products from different sectors, producing environmental and economic advantages. This is also related to the fact that the OPC industry is highly polluting. Alkali-activated precursors and cement are considered alternative and eco-friendly binders [232,236,237], usually resulting in concretes with lower CO_2_ emissions than OPC-based concrete (around 10%, according to [238]). The accurate determination of the environmental impact associated with these binders—and consequently compare it with those associated with OPC—would require a detailed analysis which will not be addressed in this paper; nonetheless, further details can be found in [239,240,241].

### 4.2. Precursors: Rich in Aluminosilicates

**Figure 9:**
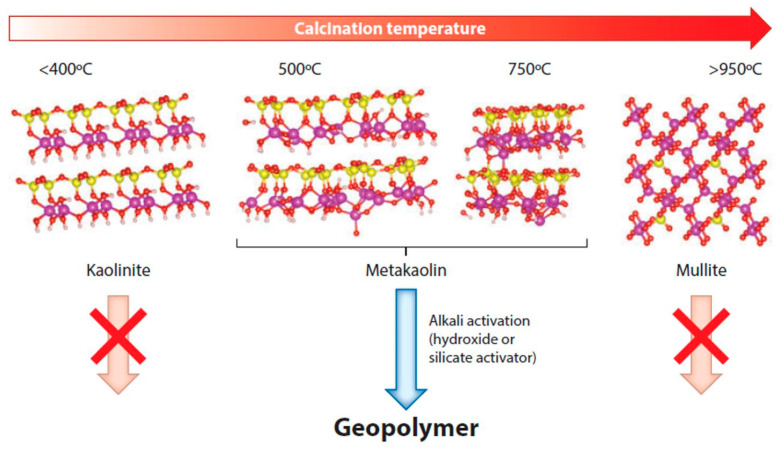
Metakaolin manufacturing process through kaolin calcination [247].

Metakaolin is one of the main precursors rich in aluminosilicates according to some authors [233,234,242]. This is due to its high reactivity and the way in which the material is obtained, which can be originated from ceramic wastes [243], or more commonly, due to calcination of clays rich in kaolinite mineral, known as kaolin [244,245]. The commercial production of metakaolin occurs with the calcination of kaolin at temperatures ranging from 500 to 800 °C, depending on the degree of crystallization and purity of the material [246]. The kaolinite present in the material undergoes a dehydroxylation reaction at around 550 °C, becoming metakaolinite. Figure 9 presents a scheme for producing metakaolin from traditional kaolin [233,247]. Burning at temperatures below 400 °C is not suitable for producing the precursor. Likewise, burning at temperatures above 950 °C is not suitable either, due to the formation of mullite that does not have the ability to be alkali activated owing to its high crystallinity and because it is not soluble in an alkaline medium [248,249].

It is interesting to note that the use of metakaolin is environmentally advantageous because metakaolin synthesis emits about 5 to 6 times less CO_2_ than the OPC production process [250]. Furthermore, kaolin can be extracted not only from mineral sources but depending on the composition it can be obtained from industrial paper waste [233]. The different sources will influence the reactivity of the metakaolin obtained, but they are ecologically more viable solutions. Another advantage is that, due to the high reactivity of metakaolin, the structure formed by alkaline activation is extremely resistant, forming a better-defined gel microstructure [233].

Regarding the disadvantages, the price of metakaolin is emphasized. Although lower than the price of OPC, it is superior to other options of precursors rich in aluminosilicates, such as fly ash [249]. Another disadvantage is related to the tendency of efflorescence of the compounds obtained by the alkaline activation of metakaolin, in general, related to the inefficiency of the chemical reaction, which can generate a whitish appearance in the concrete obtained [251,252]. The high retraction tendency of metakaolin is also mentioned, due to the chemical composition of the material, with higher levels of aluminum oxide [253].

Some examples of metakaolin-based alkali-activated cement are now presented. Hasnaoui et al. (2021) [254] evaluated the behavior of geopolymeric concretes produced with metakaolin activated by hydroxide and sodium silicate with recycled fine and coarse aggregates. The authors obtained compressive strength above 50 MPa at 28 days. Gomes et al. (2020) [255] carried out the evaluation of the mechanical properties of geopolymeric concretes produced with metakaolin activated by silicate and sodium hydroxide, containing conventional fine and coarse aggregate and steel fibers. The authors analyzed the results obtained through concepts of fracture mechanics, obtaining values compatible with application in HPC. Dias and Silva (2019) [256] evaluated the effects of the mass ratio of Na_2_O/SiO_2_ and K_2_O/SiO_2_ on the compressive strength of geopolymers produced with metakaolin without the use of silicates as activators and without the use of coarse aggregates. Albidah et al. (2020) [257] reported on the application of geopolymer concrete produced from metakaolin activated by sodium hydroxide. The authors used two types of coarse aggregates of different particle sizes, in addition to sand as a fine aggregate. The authors also used steel fiber to reinforce the geopolymer matrix, obtaining compressive strength results of approximately 58 MPa at 28 days of cure. Finally, Rocha et al. (2018) [258] evaluated the mechanical properties of geopolymers produced with metakaolin activated by hydroxide and silicate, both sodium and potassium. The authors used river washed sand as fine aggregate. The compositions evaluated by the authors reached approximately 40 MPa with 1 day of cure, showing compressive strength higher than 80 MPa after 28 days of evaluation. These results are compatible with the stipulated values for HPC.

After metakaolin, the second most used precursor rich in aluminosilicates is fly ash. As highlighted in Section 2.2, this material is obtained through the burning of mineral coal for energy production in thermoelectric plants, being one of the most produced alternative binders around the world, with an annual production of more than 900 million tons in 2019 [249]. Its characteristic is fine spherical particles, with a chemical composition based on aluminum, silicon, calcium, iron, magnesium, and carbon residues [259]. Another relevant feature is the particle size in the fly ash, ranging from <1 mm to more than 100 mm, which indicates a high specific surface area and void filling capacity. The main disadvantage of using fly ash in alkali-activated cement is the difficulty in synthesizing hardened products, due to the material curing, which must be done in thermal curing at a temperature of 65 to 90 °C to increase the material’s reactivity, which it is very low at ambient temperatures [246,260,261,262].

In the works by Luo et al. (2021) [263], the authors compared the interfacial transition zone (ITZ) behavior of two different types of paste, one based on OPC, and another based on alkali-activated cement produced with fly ash. The results obtained showed that the ITZ of the alkali-activated paste is more strongly adhered to the aggregates than the cementitious paste, contributing to the high mechanical strength of the material. Moghaddam et al. (2021) [264] evaluated the mechanical behavior of geopolymeric concrete produced with fly ash, using rubber as aggregates and incorporating steel fibers. The strength values obtained were equivalent with HPC applications. Finally, the work by Pasupathy et al. (2021) [265], investigated the durability performance of geopolymeric concretes produced with fly ash in saline environments. The authors used two particle sizes of coarse aggregate and sand as fine aggregate. As an activator solution, a combination of sodium hydroxide and silicate was used. In addition to the greater durability of geopolymer concrete, when compared to OPC-based concrete, the authors observed that the compressive strength values were compatible with HPC applications.

Other precursors rich in aluminosilicates with high application potential are calcined illite-smectite clays [266] or calcined feldspars [267,268]. In addition to these, there are volcanic ash, natural pozzolans, and metallurgical slag with low amounts of calcium [269,270,271,272]. Recently, some authors have proposed the application of industrial waste, such as chamotte, waste from the ceramic industry [243], and magnesium phosphate from the chemical industry for the production of ammonium [273]. In the studies mentioned above, the alternative precursors shown compatible performance when compared with conventional precursors (e.g., metakaolin), indicating its potential applications in HPC and UHPC. However, further investigations are required to confirm their use for these applications.

### 4.3. Precursors: Rich in Calcium

Regarding calcium-rich precursors, the main materials used are steel residues or by-products, mainly blast furnace slag. The alkaline activation of these materials results in products similar to those obtained during the hydration of OPC such as C-S-H, but with the potential to be even stronger [236,274]. Regarding calcium-rich precursors, the main materials used are steel residues or by-products, mainly blast furnace slag. The alkaline activation of these materials results in products similar to those obtained during the hydration of OPC such as C-S-H, but with the potential to be even more resistant [275]. In fact, blast furnace slag is already reactive in water, but with very low kinetics. Thus, the presence of alkaline compounds only accelerates the material’s hardening reaction [276]. This, in fact, is what motivates the use of slag as a substitute for clinker. Indeed, in the presence of this binder, which during the hydration step forms a solution rich in calcium, the slag can be activated.

**Figure 10:**
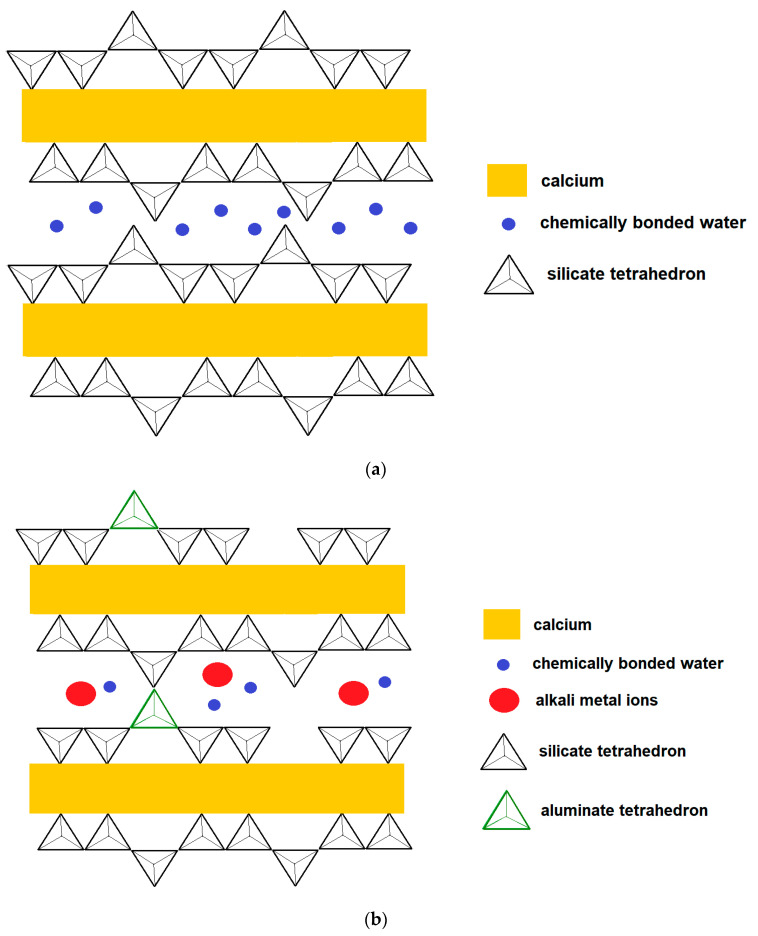
(**a**) Structure of the C-S-H; (**b**) Structure of C-A-S-H.

**Figure 11:**
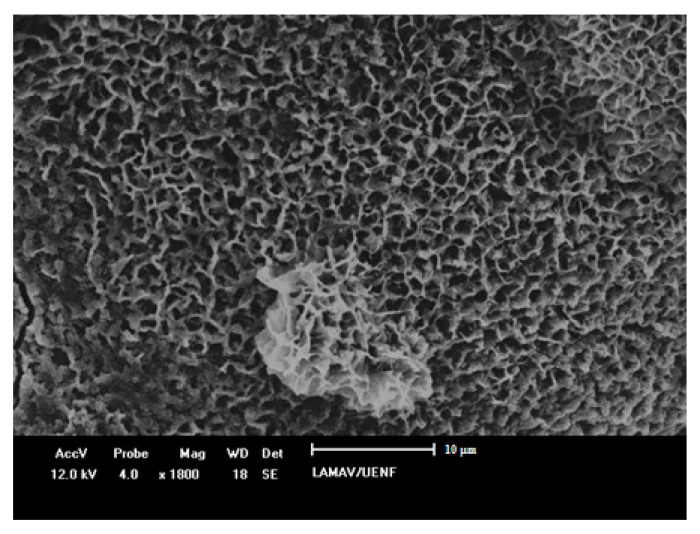
Scanning electron microscopy illustrating tobermorite obtained by activating alkali cement activated on a blast furnace slag base [236].

The products obtained by the alkaline activation of blast furnace slag in the presence of sodium hydroxide or sodium silicate are composed of hydrated calcium silicates, however, showing substitution of chemical species Si by Al. Figure 10 allows us to understand the alkaline activation process of the slag, comparing it to the hydration process of OPC. It is observed that the OPC hydration reaction produces large calcium chains linked to Si tetrahedrons and with the presence of chemically linked interstitial water. This structure is called the Dreiketten structure [232,233,275]. In the case of alkaline activation of blast furnace slag, very similar calcium chains are formed. However, some Si tetrahedra, attached to the calcium structure, are replaced by Al tetrahedrons, which allows the formation of cross-links between different Dreiketten chains, giving greater rigidity and strength to the formed compounds. The occurrence of cross-links chemically unbalances the compounds formed by the alkaline activation of the slag, which is why the presence of alkaline ions, preferable metals such as Na^+1^ and K^+1^, is necessary to promote the balance of charges. Other metallic ions can also occupy the interstice of the chains, as is the case of Al^+3^ e Ca^+2^. In addition to these ions, there is the presence of chemically bound interstitial water in the structure of this material, which is called C-A-S-H or tobermorite [277,278], to differentiate it from hydrated OPC products, usually nomenclated as C-S-H. The microscopic appearance of tobermorite is illustrated in Figure 11.

The main disadvantages of the application of blast furnace slag as a precursor in alkali-activated cement are related to the loss of workability in the material in the fresh state. Marvila et al. (2021) [236] evaluated the rheological aspects of HPC produced with alkali cement activated on the basis of blast furnace slag and sodium hydroxide solution. The authors observed that in smaller amounts of sodium, up to 7.5% Na_2_O, the obtained material behaved rheologically similar to materials based on OPC. However, at levels above 10% of Na_2_O, the materials behaved rheologically as fluids with high initial yield stress and dynamic viscosity, impairing the applicability of the material. However, this characteristic is not so relevant, because, as aforementioned, blast furnace slag is reactive to lower levels of alkalinity, enabling the application of the material with lower amounts of sodium.

It is worth highlighting some recent studies that proved the viability of using blast furnace slag as a precursor of alkali-activated cement. For example, Chen et al. (2021) [279] verified the effects of alkaline solution dosage on the properties of materials produced with alkali-activated blast furnace slag, obtaining compressive strength values above 60 MPa after 1 day of cure and above 100 MPa at 28 days, with results equivalent to the production of UHPC. He et al. (2021) [280] also produced materials based on alkali-activated cement from blast furnace slag, evaluating the influence of hydrated lime as an activated agent for the material. The results obtained were above 40 MPa after three days of curing and above 70 MPa after 28 days, with respect to compressive strength. Therefore, the feasibility of applying blast furnace slag as a precursor for alkali-activated materials is proven. Other examples of calcium-rich precursors with potential for application in alkali-activated cement are gypsum desulfurization waste (FGD), cellulosic paper sludge residues and marble residues, which are also amorphous and fine.

Another possibility of alkali-activated cement widely used by some authors is the combined application of calcium-rich and aluminosilicate-rich precursors, as is the case reported by Neupane and Hadigheh (2021) [281]. These authors produced an HPC with alkali-activated cement using silica fume and blast furnace slag, also considering two types of fine aggregate (medium and fine sand) and two types of coarse aggregate, with different particle sizes. The compressive strength results obtained were higher than 30 MPa after 7 days of curing and higher than 50 MPa after 28 days, proving the application of the material as an HPC. Kotop et al. (2021) [282] evaluated the engineering properties of concrete obtained from alkali-activated cement of fly ash and calcium-rich slag, using the application of nanoclays and carbon nanotubes. The compressive strength results obtained reached 60 MPa at 28 days, proving the viability of this type of material for HPC application. The work by Mahmood et al. (2020) [283] also evaluated the mechanical properties of concrete produced with alkali-activated cement-based on fly ash and blast furnace slag, using coarse and fine aggregates and alkaline solution based on hydroxide and sodium silicate. The results obtained for compressive strength at 28 days are above 50 MPa.

### 4.4. Activator Solution

The activating solution is produced by dissolving hydroxide and/or silicate of some alkaline metal in water as well as varying parameters of molarity and mass concentration. In general, the most used activators are sodium and potassium hydroxide, in addition to sodium silicate [258]. They play the same role as water in the hydration reaction of OPC, that is, starting the chemical reaction process of hardening the binder.

Some authors highlight the importance of using activated agents obtained by dissolving silicates since, in the alkaline activation of precursors rich in aluminosilicates, alumina dissolves before silica [232]. Thus, the use of silicates increases the reaction kinetics because it already provides, in a faster way, the necessary silica species for the activation reaction [233,275]. However, it favors the occurrence of efflorescence. In the case of alkaline activation of calcium-rich precursors, the need for the formation of tobermorite (C-A-S-H).

One of the disadvantages of using silicates is the high cost associated with this material. This highlights the need for alternative activated agents, which, in addition to reducing the cost of alkali-activated cement, contribute to the sustainable development of this type of HPC and UHPC, as indicated by Mendes et al. (2021) [284]. It is suggested, for example, the use of activated rice husk ash, glass waste, and silica fume.

## 5. Conclusions and Suggestion for Future Work

The main objective of this review article is to evaluate relevant concepts related to precursor materials used for the production of HPC and UHPC. Although there is no normative definition, these concretes can be understood as materials with high mechanical strength, good workability parameters, and high durability. After consulting the bibliography, a limit of 50 MPa for HPC and 100 MPa for UHPC was established, analyzing the compressive strength at 28 days.

Initially dealing with classic HPC and UHPC, which generally uses the same construction materials as conventional concrete, it was observed that the main type of OPC used is one that is richer in clinker and with few mineral additions. This is necessary so that pozzolans with superior quality and reactivity than those used in OPC production are used in the production of HPC and UHPC. From a chemical and mineralogical point of view, it is preferable to use OPCs rich in C_3_S and C_2_S, that is, with low levels of oxides of Al_2_O_3_ and Fe_2_O_3_.

Regarding mineral additives, the most used are pozzolanic ones, such as fly ash and silica fume, although there are HPC and UHPC applications using blast furnace slag. Silica fume is the ideal pozzolan due to its high specific surface, high amorphism content, and high presence of SiO_2_ (>95%). This promotes the occurrence of pozzolanic reactions in a more intense way. Regarding aggregates, greater care must be taken when applying them in HPC and UHPC than with aggregates used for CC. These precautions are related to the degree of packaging, chemical composition, and shape of the grains.

For the production of HPC and UHPC, it is essential to use chemical additives, especially shrinkage mitigators and superplasticizers that allow the reduction of the w/c factor without loss of workability. The 3rd generation of superplasticizers, which work due to the electrical repulsion effect and the steric effect, are the most used for these applications.

Due to the low tensile strength and lack of ductility of HPC and UHPC, some authors proposed the inclusion of fibers for the formation of composites. The main type used is steel, followed by carbon, glass, polymeric and natural, such as sisal. The fiber improves mechanical properties but reduces workability properties due to the densification of the cement matrix. As a result, fiber contents must be studied to avoid harming the behavior of HPC and UHPC.

Another new perspective on the application of HPC and UHPC emerged due to the environmental problems generated by the production of OPC clinker. Thus, the use of alkali-activated cement, with the possibility of using waste and by-products as a binder, became a reality. The alkali-activated cement is produced through a precursor, rich in calcium (usually with >20% CaO) such as blast furnace slag, or rich in aluminosilicates, giving rise to geopolymers. The main precursors of this last group are metakaolin and low-calcium fly ash. The main difference between the high-calcium and low-calcium precursors is that the former presents a higher reaction rate due to the higher solubility of calcium in comparison with silica and alumina, generally leading to higher mechanical strength at early ages compared with the latter. In addition, high-calcium precursors form calcium aluminosilicate hydrate (C-A-S-H) as the binding phase, while low-calcium precursors form 3D sodium/potassium aluminosilicate hydrate (N/K-A-S-H) frameworks. The last component used is the alkaline solution, generally based on sodium and/or potassium hydroxides or silicates. The results obtained, both in the fresh and hardened state, are compatible with HPC and UHPC applications. Besides, alkali-activated HPC and UHPC composites tend to present higher durability when compared with OPC-based composites.

In general, HPC and UHPC produced with OPC (and mineral admixtures) are easier to produce in practice because they do not require the manipulation of highly alkaline materials. In turn, those produced with alkali-activated materials generally have a lower environmental impact.

Finally, some perspectives for future work are highlighted:

- Further standardization of fiber application methodologies in HPC and UHPC;
- Development of HPC and UHPC with other natural, renewable, and more economical fibers, such as piassava, açaí, guaruman, and pineapple fibers;
- Development of alkali-activated cement dosage methodologies for application in HPC and UHPC;
- Application of other agro-industrial residues and by-products as precursors of alkali-activated cement, such as sugarcane bagasse ash and rice husk ash;
- Research on mechanisms to improve the workability and aspects of alkali-activated cement without loss of mechanical strength;
- Development of activated agents that are more ecological than sodium and/or potassium hydroxides and silicates used for the application of alkali-activated cement, such as those based on glass residue and rice husk ash.

## Figures and Tables

**Table 1:** Properties of different types of concrete.

Concrete	Abbreviation	Compressive Strength (MPa)	w/b Ratio	Workability (mm)	Cement Consumption (kg/m^3^)
Conventional	CC	20–50	0.45–0.65	NA	260–380
High Strength	HSC	55–100	NA	NA	400–700
High Performance	HPC	55–100 50–100	<0.4	455–810 (slump flow)	400–700
Ultra-High Performance	UHPC	>100 >120 >150	0.2–0.3	>260 (flow table without drops)	800–1000

NA—Not applicable.

**Table 2:** Chemical composition of Ordinary Portland Cement (OPC) used for HPC and UHPC.

CaO (%)	SiO_2_ (%)	Fe_2_O_3_ (%)	Al_2_O_3_ (%)	SO_3_ (%)	MgO (%)	Loss on Ignition (%)	Reference
62.91	20.34	4.58	4.47	2.58	1.24	3.27	[70]
61.33	21.01	3.12	6.40	2.30	3.02	-	[61]
63.62	19.70	2.93	-	-	1.28	-	[71]
66.45	17.84	3.58	4.26	4.10	2.14	-	[72]
63.07	19.38	3.28	4.58	3.50	2.79	1.54	[3]
64.62	20.18	3.24	4.98	3.15	1.98	2.59	[12]
62.60	20.60	3.20	5.10	3.60	3.00	-	[73]
67.97	16.19	3.79	3.59	4.05	1.71	0.51	[56]
71.22	14.80	3.48	4.54	4.11	-	4.02	[50]
68.91	15.74	4.80	3.18	3.80	2.00	1.00	[1]
62.90	18.90	2.80	3.70	3.10	4.20	3.20	[53]
62.15	20.95	3.80	4.85	2.00	3.10	-	[74]

**Table 3:** Physical properties of OPC used in HPC and UHPC.

Density (g/cm^3^)	Blaine Fineness (cm^2^/g)	Retained in Sieve #200 (%)	Reference
-	3600	-	[71]
3.10	3600	-	[72]
3.12	4430	0.20	[13]
3.15	3500	-	[73]
-	-	2.00	[56]
3.06	-	1.38	[51]
3.09	4070	-	[53]
3.15	-	1.80	[12]

**Table 4:** Compressive strength results obtained with the use of various water reducing additives. Source: [184].

Composition	Compressive Strength after 1 Day (MPa)	Compressive Strength after 3 Days (MPa)	Compressive Strength after 28 Days (MPa)
Reference	7.1 (100%)	19.57 (100%)	40.53 (100%)
1st generation	12.32 (174%)	31.89 (163%)	57.55 (142%)
1st generation (naphthalene)	12.81 (180%)	32.42 (166%)	51.70 (128%)
Polyfunctional	13.76 (194%)	29.01 (148%)	46.15 (114%)
Polyoxyethylene derivatives of polymethacrylic acid (PAA)	22.53 (331%)	46.38 (237%)	62.81 (155%)
Copolymer based on polyether carboxylates (PCE)	19.62 (276%)	45.01 (230%)	65.72 (162%)

**Table 5:** Properties of fibers used in HPC and UHPC. Source: [193,194,195,196,197,198,199].

Fibers	Diameter (mm)	Density (g/cm^3^)	Modulus of Elasticity (GPa)	Tensile Strength (MPa)	Elongation (%)
Matrix (comparison)	-	2.7	10–45	3.5–8	0.02
Steel	5–500	7.84	200	500–2580	0.5–3.5
Carbon	5	1.9	65–135	2600	1
Glass	9–15	2.6	70–80	2000–4000	2–4.5
Polypropylene	20–200	0.9	164	500–750	9
Polyethylene	20–200	0.95	14–19.5	200–300	7.5
Asbesto	0.02–0.4	2.6–3.4	196	33000	2–3
Kevlar	10	1.45	5–17	3600	2.1–4
Cellulose	-	1.2	4	300–500	-
Sisal	10–50	1.5	15–20	800	7

## Data Availability

All the data is available within the manuscript.
